# The prognostic values of the peroxiredoxins family in ovarian cancer

**DOI:** 10.1042/BSR20180667

**Published:** 2018-09-05

**Authors:** Saisai Li, Xiaoli Hu, Miaomiao Ye, Xueqiong Zhu

**Affiliations:** Department of Obstetrics and Gynecology, the Second Affiliated Hospital of Wenzhou Medical University, Wenzhou 325027, Zhejiang, China

**Keywords:** Kaplan-Meier plotter, ovarian carcinoma, PRDXs, prognosis, The Human Protein Atlas

## Abstract

**Purpose:** Peroxiredoxins (PRDXs) are a family of antioxidant enzymes with six identified mammalian isoforms (PRDX1–6). PRDX expression is up-regulated in various types of solid tumors; however, individual PRDX expression, and its impact on prognostic value in ovarian cancer patients, remains unclear.

**Methods:** PRDXs family protein expression profiles in normal ovarian tissues and ovarian cancer tissues were examined using the Human Protein Atlas database. Then, the prognostic roles of PRDX family members in several sets of clinical data (histology, pathological grades, clinical stages, and applied chemotherapy) in ovarian cancer patients were investigated using the Kaplan–Meier plotter.

**Results:** PRDXs family protein expression in ovarian cancer tissues was elevated compared with normal ovarian tissues. Meanwhile, elevated expression of PRDX3, PRDX5, and PRDX6 mRNAs showed poorer overall survival (OS); PRDX5 and PRDX6 also predicted poor progression-free survival (PFS) for ovarian cancer patients. Furthermore, PRDX3 played significant prognostic roles, particularly in poor differentiation and late-stage serous ovarian cancer patients. Additionally, PRDX5 predicted a lower PFS in all ovarian cancer patients treated with Platin, Taxol, and Taxol+Platin chemotherapy. PRDX3 and PRDX6 also showed poor PFS in patients treated with Platin chemotherapy. Furthermore, PRDX3 and PRDX5 indicated lower OS in patients treated with these three chemotherapeutic agents. PRDX6 predicted a poorer OS in patients treated with Taxol and Taxol+Platin chemotherapy.

**Conclusion:** These results suggest that there are distinct prognostic values of PRDX family members in patients with ovarian cancer, and that the expression of PRDX3, PRDX5, and PRDX6 mRNAs are a useful prognostic indicator in the effect of chemotherapy in ovarian cancer patients.

## Introduction

Ovarian cancer is a leading cause of morbidity and mortality among women diagnosed with gynecologic malignancies, with ~22,440 new cases and 14,080 cancer-related deaths each year in the United States [1]. The high ratio of death-to-incidence for women with ovarian cancer is mainly due to late-stage diagnosis. Importantly, many patients with ovarian cancer remain without symptoms until the disease reaches an advanced stage and it is then exceedingly difficult to treat [2]. Even with advances in diagnostic techniques, the 5-year survival rate is only ~30% [3,4]. Therefore, the identification of novel prognostic biomarkers is of critical importance and will contribute to improving the clinical outcome of ovarian cancer patients.

Peroxiredoxins (PRDXs), a family of antioxidant enzymes, are composed of six identified mammalian isoforms (PRDX1, PRDX2, PRDX3, PRDX4, PRDX5, and PRDX6) [5]. The primary role of these proteins is to protect cells from oxidative damage induced by cellular reactive oxygen species (ROS), which have been implicated in various cellular signaling pathways and the pathogenesis of diseases [6–8]. Moreover, PRDXs are frequently involved in the regulation of a series of reductant–oxidant-sensitive cellular processes, such as cell proliferation, apoptosis, and cell signaling [9–11].

Over the past several years, numerous studies have documented that PRDX expression was up-regulated in various types of solid tumors [12–17]. However, PRDXs may play dichotomous roles in the development of cancers, as either oncogenes or tumor suppressors [18]. The significance of these genes in the prognosis of malignant tumors is still complicated and in many ways contradictory [13,19–27]. Furthermore, individual PRDX expression and their impact on prognosis in ovarian cancer patients have received little research [28,29]. In the present study, we aimed to comprehensively explore immunohistochemistry (IHC)-based map of PRDXs family protein expression profiles in normal ovarian tissues and ovarian cancer tissues from the Human Protein Atlas (HPA) database. Furthermore, we also investigated the prognostic significance of PRDX family expression at the mRNA level in ovarian cancer patients using the Kaplan–Meier plotter (KM plotter).

## Materials and methods

### The Human Protein Atlas

The Human Protein Atlas (https://www.proteinatlas.org/) provided large amounts of transcriptomics and proteomics data in specific human tissues and was composed of Tissue Atlas, Cell Atlas, and Pathology Atlas. This database offered the cell-specific localization information across 44 different normal tissues and organs, as well as 20 most common types of cancer [30]. In addition, IHC-based protein expression patterns in normal human tissues and tumor tissues were used to generate an expression map by using data from HPA [31]. In the present study, we utilized this database to comprehensively explore the protein expression of the PRDX family genes in normal ovarian tissues and ovarian cancer tissues. We systematically screened the available immunohistochemistry images of six PRDX proteins presented in the database, and then selected representative images that showed trends toward differential expression in normal ovarian tissues and ovarian cancer tissues using an arbitrary selection criteria [32].

### The Kaplan–Meier plotter

The Kaplan–Meier plotter (http://kmplot.com/analysis/) [33] was used to investigate the correlation between individual PRDX mRNA levels and overall survival (OS) and progression-free survival (PFS) of 1816 ovarian cancer patients. The above online database can be used to evaluate the effect of 54,675 genes on survival rates for ovarian [34], breast [33], lung [35], and gastric cancer patients. Ovarian cancer patients were identified from the Gene Expression Omnibus (GEO), the Cancer Biomedical Informatics Grid (caBIG), and The Cancer Genome Atlas (TCGA) ovarian cancer datasets [34,35]. In addition, these datasets provided clinical data, including histology, grade, stage, TP53 mutation status, debulk, and applied chemotherapy for the ovarian cancer patients. A summary of the general patient characteristics was listed in Table 1. The database was integrated using gene expression information and ovarian cancer patients’ survival data. In order to analyze the prognostic significance of a particular gene, the selected ovarian cancer samples were divided into ‘low’ and ‘high’ according to gene mRNA expression using the auto select best cutoff value. Subsequently, the survival information (OS and PFS) for the two groups could be compared with a Kaplan–Meier survival plot. Briefly, six PRDX members (PRDX1, PRDX2, PRDX3, PRDX4, PRDX5, and PRDX6) were put into the database to acquire Kaplan–Meier survival plots. Hazard ratio (HR), 95% confidence intervals (95% CI), and log rank *P* were calculated and presented on the webpage (http://kmplot.com/analysis/index.php?p = service&cancer = ovar). *P* values of <0.05 were considered statistically significant.

**Table 1:** Clinical characteristics of ovarian cancer patients in Kaplan–Meier plotter

Variable	Overall survival (*N*)	Progress-free survival (*N*)
**Histology**		
All cancer patients	1656	1435
Serous cancer patients	1207	1104
Endometrioid cancer patients	37	51
**Pathological grades**		
I	56	37
II	324	256
III	1015	837
**Clinical stages**		
I	74	96
II	61	67
III	1044	919
IV	176	162
**TP53 mutation**		
Yes	506	483
No	94	84
**Debulk**		
Optimal	801	696
Suboptimal	536	459
**Chemotherapy**		
Contains Platin	1409	1259
Contains Taxol	793	715
Contains Taxol+Platin	776	698
**Death event**	930	978
**Median survival**	45.23 (m)	20 (m)

N, number of ovarian cancer patients with available clinical data; m, months.

## Results

**Figure 1:**
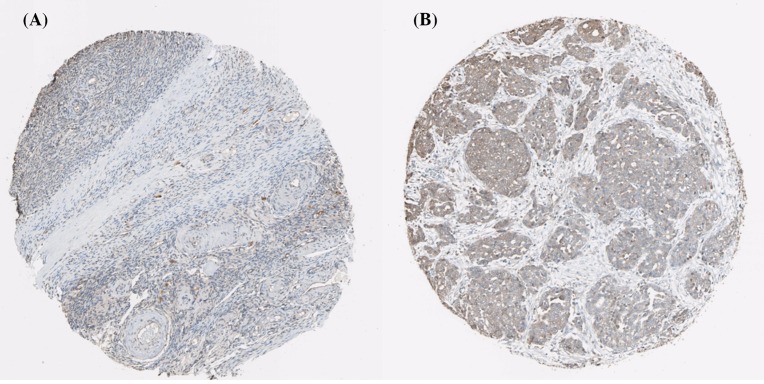
Comparison of the expression profile of PRDX1 in normal ovarian tissues and ovarian cancer tissues Representative immunohistochemistry images of PRDX1 protein expression in normal ovarian tissues (**A**) and ovarian cancer tissues (**B**). Images were downloaded from the Human Protein Atlas database (http://www.proteinatlas.org/).

**Figure 2:**
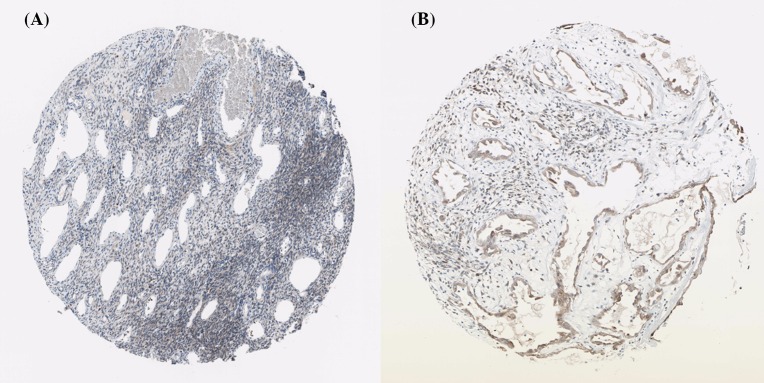
Comparison of the expression profile of PRDX2 in normal ovarian tissues and ovarian cancer tissues Representative immunohistochemistry images of PRDX2 protein expression in normal ovarian tissues (**A**) and ovarian cancer tissues (**B**). Images were downloaded from the Human Protein Atlas database (http://www.proteinatlas.org/).

**Figure 3:**
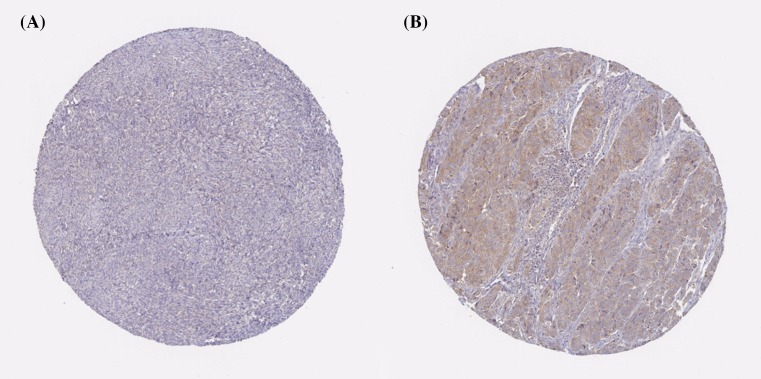
Comparison of the expression profile of PRDX3 in normal ovarian tissues and ovarian cancer tissues Representative immunohistochemistry images of PRDX3 protein expression in normal ovarian tissues (**A**) and ovarian cancer tissues (**B**). Images were downloaded from the Human Protein Atlas database (http://www.proteinatlas.org/).

**Figure 4:**
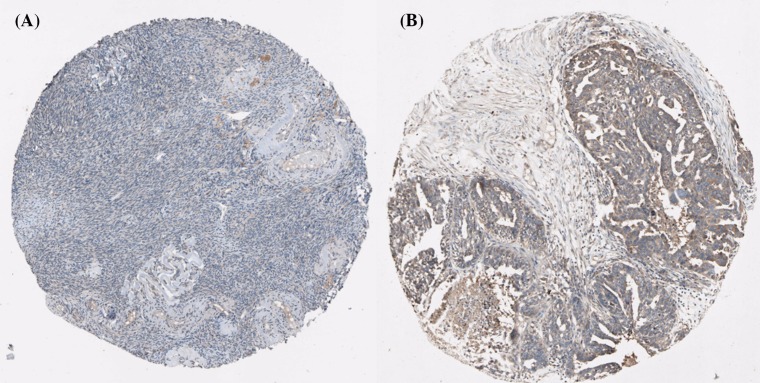
Comparison of the expression profile of PRDX4 in normal ovarian tissues and ovarian cancer tissues Representative immunohistochemistry images of PRDX4 protein expression in normal ovarian tissues (**A**) and ovarian cancer tissues (**B**). Images were downloaded from the Human Protein Atlas database (http://www.proteinatlas.org/).

**Figure 5:**
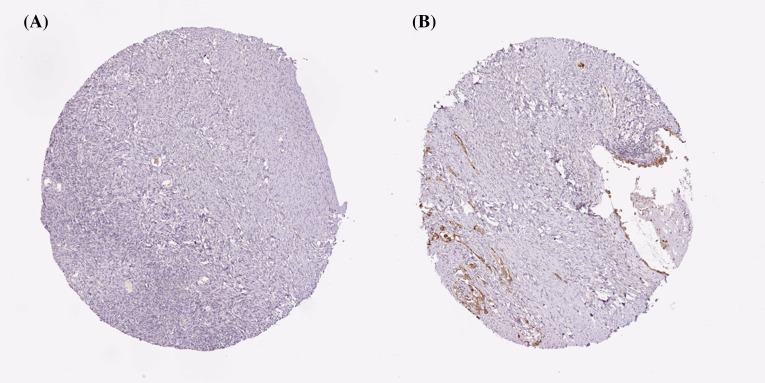
Comparison of the expression profile of PRDX5 in normal ovarian tissues and ovarian cancer tissues Representative immunohistochemistry images of PRDX5 protein expression in normal ovarian tissues (**A**) and ovarian cancer tissues (**B**). Images were downloaded from the Human Protein Atlas database (http://www.proteinatlas.org/).

**Figure 6:**
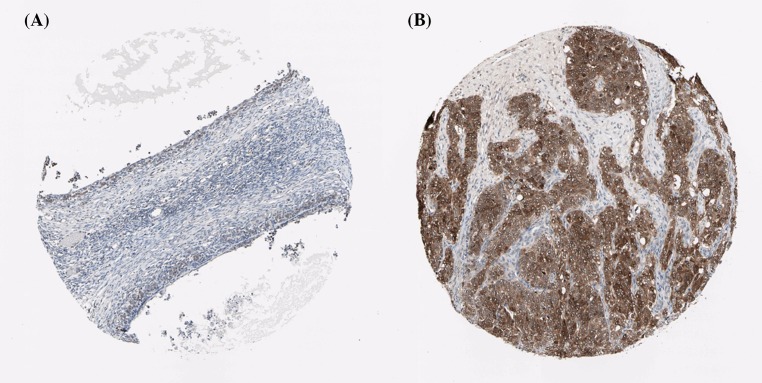
Comparison of the expression profile of PRDX6 in normal ovarian tissues and ovarian cancer tissues Representative immunohistochemistry images of PRDX6 protein expression in normal ovarian tissues (**A**) and ovarian cancer tissues (**B**). Images were downloaded from the Human Protein Atlas database (http://www.proteinatlas.org/).

Using the Human Protein Atlas database, we first analyzed the PRDXs protein expression in normal ovarian tissues and ovarian cancer tissues to determine the clinical relevance of PRDXs expression. As shown in Figure 1, we discovered that stroma cells had negative PRDX1 staining in normal ovarian tissues. In comparison, among 12 cases of ovarian cancer tissues examined, medium staining of PRDX1 was detected in the majority of cancerous tissues (ten cases), while the rest two cases had low PRDX1 staining. For PRDX2, stroma cells presented medium staining in normal ovarian tissues. Using the same antibody, there were 2 cases of high, 4 cases of medium, and 4 cases of low PRDX2 staining among the examined 11 ovarian cancer tissues (Figure 2). We found that PRDX3 protein expression was not detected in normal ovarian tissues. However, among the examined 9 ovarian cancer tissues, 8 cases had medium PRDX3 staining and 1 case had low PRDX3 staining (Figure 3). Considering PRDX4, the data showed that stroma cells had low PRDX4 staining in normal ovarian tissues. In comparison, we found that among the detected 10 ovarian cancer tissues, there were 8 cases of medium and 2 cases of low PRDX4 staining (Figure 4). Meanwhile, images of tissue staining by IHC showed that stroma cells had negative PRDX5 expression in normal ovarian tissues; however, there were 1 case of high, 9 cases of medium and 1 case of low PRDX5 staining among 12 cases of ovarian cancer tissues examined (Figure 5). Furthermore, we found that stroma cells had low PRDX6 staining in normal ovarian tissues. In comparison, there were 12 cases of ovarian cancer tissues tested, with 1 case of high, 5 cases of medium and 4 cases of low PRDX6 staining (Figure 6).

**Figure 7:**
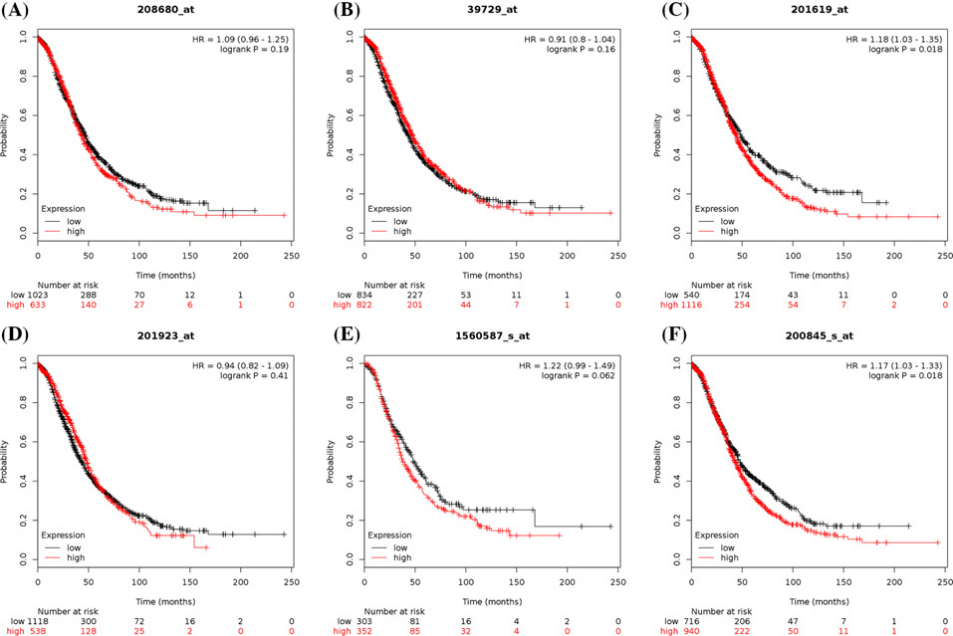
The prognostic value of peroxiredoxin (PRDX) family expression in overall survival (OS) of all ovarian cancer patients (**A**) OS curves were plotted for PRDX1 (*n*=1656). (**B**) OS curves were plotted for PRDX2 (*n*=1656). (**C**) OS curves were plotted for PRDX3 (*n*=1656). (**D**) OS curves were plotted for PRDX4 (*n*=1656). (**E**) OS curves were plotted for PRDX5 (*n*=655). (**F**) OS curves were plotted for PRDX6 (*n*=1656).

**Figure 8:**
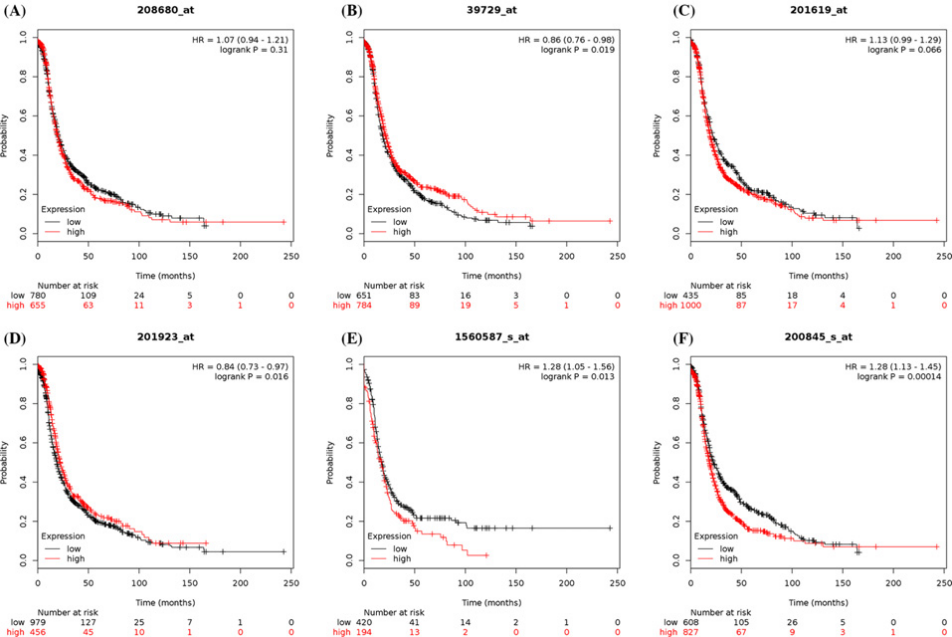
The prognostic value of peroxiredoxin (PRDX) family expression in progression-free survival (PFS) of all ovarian cancer patients (**A**) PFS curves were plotted for PRDX1 (*n*=1435). (**B**) PFS curves were plotted for PRDX2 (*n*=1435). (**C**) PFS curves were plotted for PRDX3 (*n*=1435). (**D**) PFS curves were plotted for PRDX4 (*n*=1435). (**E**) PFS curves were plotted for PRDX5 (*n*=614). (**F**) PFS curves were plotted for PRDX6 (*n*=1435).

To further examine PRDX family expression at the mRNA level, we also investigated the prognostic significance of individual PRDX family expression in ovarian cancer patients using the KM plotter. In the present study, all six PRDX members could be found in Kaplan–Meier OS and PFS information at www.kmplot.com. The prognostic value of PRDX1 mRNA expression was first accessed in the database. The desired Affymetrix ID for PRDX1 is 208680_at. OS curves (*n*=1656; Figure 7A) and PFS curves (*n*=1435; Figure 8A) were plotted for all ovarian cancer patients. As shown in Table 2, high mRNA expression of PRDX1 showed a null association with OS or PFS among all ovarian cancer patients, serous ovarian cancer patients, and endometrioid ovarian cancer patients. To further access the relationship between individual PRDXs and other clinicopathological features, the association with pathological grade, clinical stage, and chemotherapy of ovarian carcinoma patients was examined. Analysis indicated that a high expression of PRDX1 was correlated with a better OS in grade I or II ovarian cancer patients. In addition, PRDX1 also predicted a better PFS in grade I ovarian cancer patients. However, the clinical stage results showed that high levels of PRDX1 mRNA were associated with a poorer PFS in stages I and II ovarian cancer patients. Furthermore, increased PRDX1 mRNA expression was not correlated with OS or PFS among all ovarian cancer patients treated with Platin, Taxol, and Taxol+Platin chemotherapy.

**Table 2:** The prognostic value of PRDX1 mRNA expression in ovarian cancer

	Overall survival			Progress-free survival		
	Cases	HR (95% CI)	*P*-value	Cases	HR (95% CI)	*P*-value
**Histology**						
All cancer patients	1656	1.09 (0.96–1.25)	0.19	1435	1.07 (0.94–1.21)	0.31
Serous cancer patients	1207	1.15 (0.98–1.36)	0.092	1104	0.91 (0.78–1.06)	0.24
Endometrioid cancer patients	37	2.1 (0.35–12.6)	0.41	51	0.3 (0.07–1.29)	0.086
**Pathological grades**						
I	56	0.25 (0.09–0.7)	0.0044*	37	0.14 (0.04–0.42)	5.4e−05*
II	324	0.71 (0.52–0.97)	0.028*	256	0.75 (0.53–1.05)	0.094
III	1015	0.92 (0.75–1.12)	0.4	837	0.91 (0.77–1.09)	0.3
**Clinical stages**						
I and II	135	0.52 (0.18–1.5)	0.22	163	2.12 (1.06–4.27)	0.03*
III and IV	1220	1.15 (0.98–1.35)	0.084	1081	0.89 (0.76–1.04)	0.15
**Chemotherapy**						
Contains Platin	1409	0.91 (0.77–1.07)	0.26	1259	1.08 (0.95–1.23)	0.24
Contains Taxol	793	1.17 (0.96–1.41)	0.12	715	0.9 (0.76–1.07)	0.23
Contains Taxol+Platin	776	1.16 (0.95–1.41)	0.14	698	0.9 (0.75–1.07)	0.24

*P<0.05.

Subsequently, the prognostic significance of PRDX2 expression was determined in the database (Figures 7B and 8B, and Table 3). The desired Affymetrix ID for PRDX2 is 39729_at. Highly expressed PRDX2 mRNA was not found to be correlated with OS in all histological subtypes of ovarian cancer patients. However, elevated mRNA expression of PRDX2 was significantly correlated with better PFS for all ovarian cancer patients and serous ovarian cancer patients. In addition, high expression of PRDX2 mRNA was correlated with a better PFS in grade II or III ovarian cancer patients. Furthermore, increased expression of PRDX2 in stages III and IV ovarian cancer patients was related to a better PFS. High PRDX2 expression was not linked to OS among all ovarian cancer patients treated with Platin, Taxol, and Taxol+Platin chemotherapy, but results among these three chemotherapeutic agents showed a better PFS in all ovarian cancer patients.

**Table 3:** The prognostic value of PRDX2 mRNA expression in ovarian cancer

	Overall survival			Progress-free survival		
	Cases	HR (95% CI)	*P*-value	Cases	HR (95% CI)	*P*-value
**Histology**						
All cancer patients	1656	0.91 (0.8–1.04)	0.16	1435	0.86 (0.76–0.98)	0.019*
Serous cancer patients	1207	0.89 (0.77–1.04)	0.15	1104	0.73 (0.63–0.85)	2.6e−05*
Endometrioid cancer patients	37	0.33 (0.06–1.98)	0.2	51	0.44 (0.17–1.11)	0.075
**Pathological grades**						
I	56	0.67 (0.26–1.73)	0.4	37	0.4 (0.13–1.21)	0.094
II	324	0.81 (0.6–1.09)	0.17	256	0.62 (0.46–0.84)	0.0019*
III	1015	1.09 (0.9–1.31)	0.37	837	0.74 (0.63–0.88)	0.00041*
**Clinical stages**						
I and II	135	1.97 (0.89–4.34)	0.087	163	1.35 (0.76–2.4)	0.3
III and IV	1220	1.16 (0.98–1.37)	0.087	1081	0.76 (0.66–0.88)	0.00018*
**Chemotherapy**						
Contains Platin	1409	0.89 (0.78–1.03)	0.11	1259	0.84 (0.74–0.96)	0.01*
Contains Taxol	793	0.89 (0.74–1.08)	0.25	715	0.77 (0.65–0.91)	0.0026*
Contains Taxol+Platin	776	1.1 (0.89–1.36)	0.39	698	0.77 (0.65–0.92)	0.0034*

*P<0.05.

For PRDX3, its desired Affymetrix ID is 201619_at (Figures 7C and 8C, and Table 4). Increased PRDX3 mRNA expression was found to be related to a poorer OS in all ovarian cancer patients and serous ovarian cancer patients, but not in endometrioid cancer patients. However, high mRNA expression of PRDX3 showed no effect on PFS in different histological types of ovarian cancer patients. High levels of PRDX3 mRNA were correlated to a poorer OS in grade III ovarian cancer patients, while PRDX3 predicted a better PFS in 37 patients with grade I ovarian cancer. Furthermore, the clinical stage results showed that high expression of PRDX3 mRNA was associated with a poorer OS in stages III and IV ovarian cancer patients. Additionally, increased PRDX3 mRNA expression was associated with poorer OS in all ovarian cancer patients treated with Platin, Taxol, and Taxol+Platin chemotherapy; PRDX3 also showed a poor PFS in all patients treated with Platin chemotherapy.

**Table 4:** The prognostic value of PRDX3 mRNA expression in ovarian cancer

	Overall survival			Progress-free survival		
	Cases	HR (95% CI)	*P*-value	Cases	HR (95% CI)	*P*-value
**Histology**						
All cancer patients	1656	1.18 (1.03–1.35)	0.018*	1435	1.13 (0.99–1.29)	0.066
Serous cancer patients	1207	1.18 (1.01–1.37)	0.036*	1104	0.93 (0.81–1.08)	0.35
Endometrioid cancer patients	37	0.36 (0.06–2.18)	0.25	51	0.66 (0.26–1.67)	0.38
**Pathological grades**						
I	56	0.41 (0.16–1.07)	0.06	37	0.34 (0.11–1.03)	0.047*
II	324	1.21 (0.88–1.66)	0.24	256	0.79 (0.57–1.08)	0.14
III	1015	1.19 (1.01–1.4)	0.039*	837	1.15 (0.96–1.37)	0.13
**Clinical stages**						
I and II	135	1.53 (0.7–3.35)	0.29	163	1.24 (0.7–2.19)	0.46
III and IV	1220	1.28 (1.1–1.48)	0.0012*	1081	0.92 (0.79–1.06)	0.24
**Chemotherapy**						
Contains Platin	1409	1.16 (1–1.35)	0.047*	1259	1.22 (1.06–1.4)	0.0042*
Contains Taxol	793	1.26 (1.05–1.52)	0.015*	715	1.14 (0.94–1.39)	0.18
Contains Taxol+Platin	776	1.27 (1.05–1.53)	0.014*	698	1.13 (0.93–1.38)	0.21

*P<0.05.

The prognostic significance of the expression of PRDX4 was further determined in the database (Figures 7D and 8D, and Table 5). The desired Affymetrix ID for PRDX4 is 201923_at. Overexpression of PRDX4 mRNA was correlated with a favorable OS for endometrioid cancer patients. In addition, PRDX4 predicted better PFS in all ovarian cancer patients, serous ovarian cancer patients, and endometrioid cancer patients. Elevated PRDX4 mRNA expression was associated with a better PFS in grade II or III ovarian cancer patients. The clinical stage results showed that high mRNA expression of PRDX4 was related to a positive OS in stages I and II ovarian cancer patients. Furthermore, PRDX4 also predicted a better PFS in stages III and IV ovarian cancer patients, while PRDX4 presented a poorer OS in stages III and IV ovarian cancer patients. In addition, elevated PRDX4 mRNA expression was related to better PFS in all ovarian cancer patients treated with Platin, Taxol, and Taxol+Platin chemotherapy, while PRDX4 in all patients treated with these three chemotherapeutic agents showed no correlation with OS.

**Table 5:** The prognostic value of PRDX4 mRNA expression in ovarian cancer

	Overall survival			Progress-free survival		
	Cases	HR (95% CI)	*P*-value	Cases	HR (95% CI)	*P*-value
**Histology**						
All cancer patients	1656	0.94 (0.82–1.09)	0.41	1435	0.84 (0.73–0.97)	0.016*
Serous cancer patients	1207	0.88 (0.76–1.03)	0.11	1104	0.77 (0.64–0.92)	0.0033*
Endometrioid cancer patients	37	0.1 (0.01–0.88)	0.01*	51	0.2 (0.07–0.53)	0.00039*
**Pathological grades**						
I	56	0.47 (0.18–1.21)	0.11	37	0.19 (0.02–1.47)	0.076
II	324	0.74 (0.53–1.02)	0.067	256	0.68 (0.5–0.92)	0.012*
III	1015	0.9 (0.75–1.07)	0.24	837	0.76 (0.65–0.91)	0.0018*
**Clinical stages**						
I and II	135	0.34 (0.15–0.77)	0.0069*	163	0.61 (0.32–1.15)	0.12
III and IV	1220	1.29 (1.09–1.53)	0.0026*	1081	0.79 (0.66–0.94)	0.0072*
**Chemotherapy**						
Contains Platin	1409	0.87 (0.75–1.02)	0.095	1259	0.82 (0.71–0.94)	0.0053*
Contains Taxol	793	1.2 (0.97–1.47)	0.087	715	0.77 (0.64–0.93)	0.0067*
Contains Taxol+Platin	776	1.22 (0.99–1.5)	0.067	698	0.75 (0.62–0.91)	0.0037*

*P<0.05.

Next, the prognostic significance of PRDX5 expression in the database was determined (Figures 7E and 8E, and Table 6). The desired Affymetrix ID for PRDX5 is 1560587_s_at. High PRDX5 mRNA expression was associated with a poorer OS for serous ovarian cancer patients and endometrioid cancer patients. PRDX5 also predicted a poor PFS for all ovarian cancer patients and endometrioid cancer patients. Furthermore, increased mRNA expression of PRDX5 was found to be correlated with a poorer OS in grade II or III ovarian cancer patients and a poorer PFS in grade II ovarian cancer patients. Nevertheless, PRDX5 showed a better OS in 41 patients with grade I ovarian cancer. Further studies revealed that high PRDX5 mRNA expression was associated with a poor PFS in stages III and IV ovarian cancer patients. However, PRDX5 indicated a positive OS in stages I and II ovarian cancer patients. Elevated PRDX5 mRNA expression was correlated with poorer OS and PFS in all ovarian cancer patients treated with Platin, Taxol, and Taxol+Platin chemotherapy.

**Table 6:** The prognostic value of PRDX5 mRNA expression in ovarian cancer

	Overall survival			Progress-free survival		
	Cases	HR (95% CI)	*P*-value	Cases	HR (95% CI)	*P*-value
**Histology**						
All cancer patients	655	1.22 (0.99–1.49)	0.062	614	1.28 (1.05–1.56)	0.013*
Serous cancer patients	523	1.25 (1–1.57)	0.046*	483	0.8 (0.64–1.01)	0.057
Endometrioid cancer patients	30	7.8 (0.81–75.29)	0.036*	44	6.85 (1.52–30.91)	0.0039*
**Pathological grades**						
I	41	0.26 (0.07–0.97)	0.031*	28	0.45 (0.11–1.8)	0.25
II	162	1.66 (1.07–2.56)	0.022*	161	1.54 (1.06–2.23)	0.022*
III	392	1.45 (1.11–1.89)	0.0067*	315	0.84 (0.64–1.1)	0.2
**Clinical stages**						
I and II	83	0.33 (0.12–0.93)	0.027*	115	0.54 (0.25–1.16)	0.11
III and IV	487	1.23 (0.98–1.54)	0.076	494	1.4 (1.14–1.73)	0.0011*
**Chemotherapy**						
Contains Platin	1409	1.37 (1.09–1.73)	0.0074*	1259	1.41 (1.15–1.73)	0.00079*
Contains Taxol	793	1.44 (1.08–1.92)	0.012*	715	1.44 (1.13–1.84)	0.0033*
Contains Taxol+Platin	776	1.44 (1.08–1.91)	0.013*	698	1.45 (1.13–1.85)	0.003*

*P<0.05.

Finally, the prognostic value of the expression of PRDX6 was investigated in the database (Figures 7F and 8F, and Table 7). The desired Affymetrix ID for PRDX6 is 200845_s_at. It was found that high levels of PRDX6 mRNA were correlated to a poorer OS for all ovarian cancer patients. Meanwhile, the curves showed that high PRDX6 mRNA expression was associated with poorer PFS for all ovarian cancer patients and endometrioid cancer patients. Furthermore, high PRDX6 mRNA expression was found to be correlated with poorer OS in grade III ovarian cancer patients. Elevated expression of PRDX6 mRNA was associated with poorer PFS in stages I and II ovarian cancer patients, whereas PRDX6 in stages III and IV ovarian cancer patients displayed better PFS. Furthermore, overexpression of PRDX6 was correlated with poorer OS in all ovarian cancer patients treated with Taxol and Taxol+Platin chemotherapy, while PRDX6 indicated a poor PFS in all ovarian cancer patients treated with Platin chemotherapy.

**Table 7:** The prognostic value of PRDX6 mRNA expression in ovarian cancer

	Overall survival			Progress-free survival		
	Cases	HR (95% CI)	*P*-value	Cases	HR (95% CI)	*P*-value
**Histology**						
All cancer patients	1656	1.17 (1.03–1.33)	0.018*	1435	1.28 (1.13–1.45)	0.00014*
Serous cancer patients	1207	1.14 (0.98–1.32)	0.097	1104	0.87 (0.75–1.01)	0.059
Endometrioid cancer patients	37	0.17 (0.02–1.48)	0.066	51	3.62 (1.34–9.77)	0.0069*
**Pathological grades**						
I	56	2.08 (0.67–6.43)	0.19	37	2.02 (0.45–9.19)	0.35
II	324	1.32 (0.94–1.87)	0.11	256	0.82 (0.61–1.09)	0.17
III	1015	1.19 (1.01–1.41)	0.036*	837	0.93 (0.79–1.1)	0.41
**Clinical stages**						
I and II	135	1.72 (0.79–3.72)	0.17	163	2.76 (1.56–4.87)	0.00027*
III and IV	1220	1.15 (0.98–1.34)	0.079	1081	0.8 (0.7–0.93)	0.0025*
**Chemotherapy**						
Contains Platin	1409	1.15 (0.99–1.33)	0.06	1259	1.21 (1.06–1.38)	0.0039*
Contains Taxol	793	1.3 (1.08–1.58)	0.0063*	715	1.11 (0.94–1.32)	0.23
Contains Taxol+Platin	776	1.33 (1.08–1.63)	0.0073*	698	1.11 (0.93–1.32)	0.25

*P<0.05.

## Discussion

PRDXs, a family of antioxidant enzymes, play dominant roles in regulating cellular peroxide levels, which are essential for cell signaling and metabolism [36]. It has been demonstrated that an imbalance between the generation of ROS and PRDXs in cancer cells could cause oxidative stress and the induction of apoptosis [37]. PRDXs expression is up-regulated under oxidative stress conditions. Several investigations have suggested that overexpression of PRDXs may play dichotomous role in carcinogenesis, where they could either promote the growth of cancers or inhibit the development of cancers [18]. Although several researches of PRDXs have been published, little is known about individual PRDX expression and their impact on prognosis in ovarian cancer patients. In the present study, we first compared the PRDXs protein expression in normal ovarian tissues and in ovarian cancer tissues by using the Human Protein Atlas database, and found obviously up-regulated PRDXs protein expression in ovarian cancer tissues. Then we comprehensively accessed the prognostic significance of all the six PRDX members in patients with ovarian carcinoma by using the KM plotter database. According to our results, high levels of PRDX3, PRDX5, and PRDX6 indicated poor clinical outcomes in ovarian cancer. However, the use of PRDX1, PRDX2, and PRDX4 as a prognostic indicator in ovarian cancer needs further study.

Previous studies have shown that PRDX1 might play tumor suppressive role in breast cancers, and the anti-tumor effect of PRDX1 was regulated via c-Myc or PTEN pathways [38,39]. On the contrary, PRDX1 has a tumor promoting role in breast cancer, bladder cancer, oral squamous cell carcinoma, lung cancer, and esophageal squamous cell carcinoma through activation of NF-κB pathway, FOXO1-mediated pathway, and mTOR/p70S6K pathway [40–44]. A number of studies have addressed the association between PRDX1 expression and prognosis in several types of human cancers; however, the results are inconsistent and inconclusive. O’Leary et al. [45] observed that increased PRDX1 expression was an independent predictor of improved prognosis in estrogen receptor-positive breast cancer. In addition, low expression of PRDX1 indicated the risk of tumor progression and correlated significantly with reduced survival in oral squamous cell carcinoma [19], cholangiocarcinoma [21], esophageal squamous cell carcinoma [46], and pancreatic adenocarcinoma [23]. Meanwhile, there is also a large body of research demonstrating that overexpression of PRDX1 is a significant indicator of poor prognosis in non-small cell lung cancer [47], hilar cholangiocarcinoma [22], oral squamous cell carcinoma [20], pancreatic cancer [24], hepatocellular carcinoma [48], and rectal cancer [49]. In addition to these discrepancies, studies about the prognosis of PRDX1 in ovarian cancer are limited. Chung et al. [28] initially addressed the prognostic value of PRDX1 in ovarian cancer using proteomic analysis and further confirmed the findings using immunohistochemistry and Western blot. They observed that PRDX1 was overexpressed in most malignant ovarian tumors and was correlated with a poorer overall survival rate in patients suffering from ovarian serous cancer. Consistently, our results using HPA database showed that PRDX1 protein expression was elevated in ovarian cancer tissues, while it was not detected in normal ovarian tissue. However, further analysis via KM plotter database indicated that high levels of PRDX1 showed no effect on OS or PFS among all ovarian cancer patients, serous ovarian cancer patients, or endometrioid ovarian cancer patients. We subsequently observed that PRDX1 indicated a better OS in grade I or II patients and a favorable PFS in grade I patients; however, this gene predicted a poorer PFS in stages I and II patients. Taken together, the prognostic value of PRDX1 in ovarian cancer remains controversial and requires further study.

The tumor promoting effect of PRDX2 was demonstrated in colorectal carcinoma through up-regulation of Wnt/β-catenin pathway [50], and prostate cancer through increased activation of the androgen receptor (AR) signaling pathway [51]. The study by Raatikainen et al. [52] showed that augmented PRDX2 expression predicted a shorter biochemical recurrence-free survival and poor overall survival in prostate cancer patients. In a recent study, Peng et al. [26] observed that the expression of PRDX2 was significantly up-regulated in colorectal cancer. Furthermore, overexpression of PRDX2 was associated with colorectal cancer progression and shorter patient survival. However, Ji et al. [25] noted that the decreased expression of PRDX2 was associated with liver metastases and poorer OS in patients with colorectal cancer. In addition, previous studies demonstrated that patients exhibiting PRDX2 positive had a better prognosis than their PRDX2 negative counterparts in renal cell carcinomas [53] and astrocytic brain tumors [54]. However, few studies have focused on the relationship between PRDX2 expression and disease outcome in ovarian cancer patients. Sova et al. [55] showed that PRDX2 expression was decreased in endometriosis-associated ovarian cancer when compared with benign endometriosis and endometriotic tissue from patients with endometriosis-associated ovarian cancer endometriotic tissue. Furthermore, Li et al. [56] conducted a comparative proteomic study and found that PRDX2 expression was linearly decreased from normal ovarian tissue, benign ovarian tissue, and to ovarian cancer tissue, they therefore presumed that PRDX2 might play a suppressive role in tumor formation and progression. However, there is no further study on the prognostic value of PRDX2 in ovarian cancer. In the present study, we found that elevated PRDX2 expression was correlated with a better PFS in all ovarian cancer patients, especially in serous ovarian cancer patients. In addition, high expression of PRDX2 predicted a better PFS in grade II or III patients and stages III and IV patients. However, observation from HPA database revealed that PRDX2 protein expression was up-regulated in ovarian cancer tissues, suggesting that PRDX2 may promote the progression of ovarian cancer. Due to the conflicting results between the two publicly available databases, further investigation needs to be done to better understand PRDX2 expression and its impact on prognosis in ovarian cancer patients.

Karihtala et al. [13] found that elevated PRDX3 level was correlated with a better prognosis in patients with breast carcinoma, probably resulting from its association with the presence of progesterone and estrogen receptors. However, Chua et al. [57] showed that PRDX3 played a tumor-promoting role in breast cancers through regulation of the cell cycle and cell proliferation. Woolston et al. [27] demonstrated that high cytoplasmic expression of PRDX3 was linked with poor prognosis in breast cancer patients. In addition, Hintsala et al. [58] showed that up-regulation of PRDX3 in melanocytic skin tumors was correlated with shortened melanoma-specific survival. Furthermore, PRDX3 overexpression was strongly associated with the progression of hepatocellular carcinoma; patients with high serum PRDX3 levels had a shorter median survival time when compared to those with low serum PRDX3 level [59]. Li and co-workers have previously reported that the expression of PRDX3 was up-regulated in ovarian serous cystadenocarinoma specimens when compared with the normal ovarian epithelia [56]. In addition, Duan et al. [60] observed that the expression of PRDX3 was significantly higher in cancer tissues than the adjacent non-cancerous tissues, and high PRDX3 levels in serous ovarian carcinoma were related to poorly differentiated cancer cells, FIGO stages III and IV, which suggests that aberrant expression of PRDX3 is significantly associated with the progression of ovarian cancer. Wang et al. [61] found that PRDX3 expression was significantly higher in the platinum-resistant serous ovarian cancer, in stages III and IV, and in moderately and poorly differentiated ovarian cancer tissues compared with their platinum-sensitive counterparts, they therefore concluded that PRDX3 was associated with drug resistance in ovarian cancer. However, there are no reports concerning the prognostic value of either PRDX3 protein or mRNA in ovarian cancer. In this study, immunohistochemistry analysis via HPA database showed that the expression of PRDX3 protein in ovarian cancer tissues was significantly up-regulated compared with normal ovarian tissues. In addition, by using the KM plotter database, we found that increased PRDX3 expression was correlated with poorer OS in all ovarian cancer patients, especially for serous ovarian cancer patients. In addition, high PRDX3 levels predicted a poorer OS in grade III patients, stages III and IV patients, while PRDX3 predicted a better PFS in 37 patients with grade I ovarian cancer; this finding might have been due to the small and unbalanced sample sizes. Based on our study, PRDX3 may predict a dismal prognosis in patients with ovarian cancer, particularly in those with poor differentiation and late-stage serous ovarian cancer.

Wei et al. [62] showed that sulfiredoxin (Srx) preferentially interacted with PRDX4 and the Srx–PRDX4 axis led to the maintenance of lung tumor phenotype *in vitro* and metastasis formation *in vivo* via AP-1/MMP-9 and MAPK signaling pathway. Several previous studies reported that high expression of PRDX4 demonstrated an unfavorable prognosis in colorectal cancer [63], lung squamous cell carcinoma [64], oral cavity squamous cell carcinoma [65], and urinary bladder carcinoma [66]. However, there are also a few studies showing that PRDX4 is a favorable prognostic marker in malignancies. Karihtala et al. [13] demonstrated that PRDX4 was overexpressed in progesterone receptor positive patients and correlated to a better prognosis in patients with breast carcinoma. The study of Hintsala et al. [58] observed that cytoplasmic PRDX4 expression might play a protective role in malignant melanomas and offer a better prognosis. However, until the initiation of the current study, there is limited data about the prognostic value of PRDX4 in ovarian cancer. A study evaluating 68 invasive ovarian carcinomas using immunohistochemistry reported that the mean survival in patients with higher cytoplasmic PRDX4 expression was longer than for patients with lower PRDX4 expression; these authors therefore proposed that PRDX4 was associated with a better prognosis in ovarian cancer, but this was not independent of histological grade or clinical stage [29]. In the current study, we investigated the prognostic value of PRDX4 in several sets of clinical data, including histology, grade, stage, and applied chemotherapy for 1816 ovarian cancer patients. Our results showed that high PRDX4 level was related to a favorable OS for endometrioid cancer patients and a better PFS for all ovarian cancer patients, serous cancer patients, and endometrioid cancer patients. Additionally, increased expression of PRDX4 was associated with a positive OS in stages I and II patients and a better PFS in grade II or III patients, stages III and IV ovarian cancer patients. However, by comparing with public HPA database, we discovered that PRDX4 was significantly up-regulated at the protein levels in ovarian cancer tissues than that in normal ovarian tissues, implying that PRDX4 may play an essential role in the development of ovarian cancer. Due to these rather contradictory results, further research regarding the role of PRDX4 in ovarian cancer is needed.

Gerard et al. [67] demonstrated that PRDX5 promoted Graves’ disease and PRDX5 expression was directly associated with the functional status of epithelial cells. Kim et al. [68] suggested that overexpression of PRDX5 is significantly associated with malignant behavior (tumor size, depth of tumor, and lymphatic invasion) of gastric cancer. They suggested that high levels of PRDX5 enhance carcinogenicity and contribute to poor prognosis of gastric cancer. In patients with breast cancer, increased expression of PRDX5 was found to be significantly correlated with a shorter patient survival [13]. Moreover, Han et al. [12] demonstrated that the level of PRDX5 was elevated and PRDX5 expression in endometrial cancer was significantly associated with a poorer survival rate, suggesting that PRDX5 may be a clinically prognostic biomarker for the development of endometrial cancer. But so far, there is only one study that has previously reported the role of PRDX5 in ovarian cancer, and it merely revealed that high PRDX5 cytoplasmic expression was correlated with a higher stage in ovarian cancer, but did not further analyze the prognostic value of PRDX5 in ovarian cancer patients [29]. In the current study, HPA database outcomes showed that the expression of PRDX5 protein was elevated in ovarian cancer tissues, which was completely not detected in normal ovarian tissues. Using the KM plotter database, we explored the correlation of PRDX5 mRNA levels to OS and PFS of 1816 ovarian cancer patients. Our results showed that high PRDX5 expression was associated with poorer OS for serous ovarian cancer patients, endometrioid ovarian cancer patients, and grade II or III ovarian cancer patients. Furthermore, PRDX5 also predicted poor PFS for all ovarian cancer patients, endometrioid ovarian cancer patients, grade II ovarian cancer patients, and stages III and IV ovarian cancer patients. Based on previous evidence as well as our results, this gene may be a poor prognostic indicator in ovarian carcinoma patients.

Recent studies suggest that PRDX6 is a predicative biomarker for the prognosis of patients with malignant tumors; however, there is no consensus on the results. Isohookana et al. [23] reported that lack of cytoplasmic PRDX6 expression correlated with shorter disease-free survival in patients with larger pancreatic adenocarcinoma tumor size. Xu et al. [69] found that PRDX6 was highly expressed in the peri-tumoral tissues and played a critical role in inhibiting the carcinogenesis of hepatocellular carcinoma. However, Yun et al. [70,71] demonstrated that PRDX6 promoted the development of lung cancer via JAK2/STAT3 pathway, its GPx and iPLA2 activities. The study of Raatikainen et al. [52] showed that high PRDX6 expression was related to shortened biochemical recurrence-free survival and OS in prostate cancer patients after radical prostatectomy. Another study suggested that high level of PRDX6 was correlated with shorter 5-year disease-specific survival in patients with diffuse large B-cell lymphoma [72]. Nevertheless, publications about PRDX6 in ovarian cancer are limited. Karihtala et al. [29] observed that PRDX6 was overexpressed in the progression of ovarian carcinomas; however, no other studies further investigated its prognostic value in this disease. In the present study, our results using HPA database showed that increased PRDX6 protein expression in ovarian cancer tissues compared with normal ovarian tissues. By analyzing the KM plotter database, we reported for the first time that PRDX6 overexpression was linked with a poorer OS for all ovarian cancer patients and grade III ovarian cancer patients. Meanwhile, PRDX6 predicted poor PFS for all ovarian cancer patients, endometrioid ovarian cancer patients, stages I and II ovarian cancer patients. Therefore, our current results demonstrate that PRDX6 overexpression is a significant indicator of poor clinical outcome for ovarian cancer patients.

The generation of ROS has been reported to play a key role in the formation of cancer [73]. ROS scavenging by antioxidant enzymes have important implications for the efficacy and toxicity of chemotherapeutic drugs. As antioxidants, PRDXs have been shown to be related to chemotherapy drug resistance of cancers. PRDX3 expression was associated with platinum resistance in ovarian cancer, and siRNA targeting of PRDX3 triggered cisplatin–induced apoptosis in SKOV3 ovarian cancer cells through suppression of the NF–κB signaling pathway [60,61]. Furthermore, overexpression of PRDX6 attenuated cisplatin-induced apoptosis by reducing ROS levels in SKOV-3 ovarian cancer cells and led to the development of cisplatin resistance [74]. In addition, Kalinina et al. [75] demonstrated that there was a significant increase in the expression of PRDX1, PRDX3, and PRDX6 in cisplatin-resistant ovarian cancer cell lines when compared with their sensitive counterparts, implying that these isoforms might play an important role in the development of cisplatin resistance of ovarian cancer cells. Collectively, these data suggest that these isoforms may be the potential targets in cancer therapy. However, the prognostic value of PRDXs family in ovarian cancer patients treated with chemotherapy agents is unknown. In the present study, we found that high PRDX3 levels predicted a poorer OS in all patients treated with Platin, Taxol, and Taxol+Platin chemotherapy; furthermore, PRDX3 was also associated with a poor PFS in patients treated with Platin chemotherapy. Moreover, we observed that PRDX5 overexpression was related to unfavorable OS and PFS in all ovarian cancer patients treated with Platin, Taxol, and Taxol+Platin chemotherapy. PRDX6 overexpression was linked with a poorer OS in all ovarian cancer patients treated with Taxol and Taxol+Platin chemotherapy. Meanwhile, PRDX6 predicted poor PFS in all ovarian cancer patients treated with Platin chemotherapy. Taken together, these observations indicated that PRDX3, PRDX5, and PRDX6 overexpression could lead to the chemotherapy resistance in ovarian cancer and therapeutic strategies targeting these isoforms may therefore be an effective anticancer therapy for ovarian cancer. Cruz et al. [76] revealed that PRDX2 was up-regulated in drug-resistant ovarian cancer and might be a potential biomarkers for the development of chemoresistance in ovarian cancer. Sehrawat et al. [77] observed that PRDX4 expression was up-regulated in drug resistance to advanced ovarian cancer patients receiving first-line chemotherapy of paclitaxel and carboplatin, suggesting that PRDX4 may act as a candidate biomarker to predict chemotherapy response in ovarian cancer. In contrast, we found that PRDX2 and PRDX4 showed better PFS in all ovarian cancer patients treated with Platin, Taxol, and Taxol+Platin chemotherapy, implying that PRDX2 and PRDX4 predict better prognosis in ovarian cancer patients treated with these three chemotherapeutic drugs. Further studies need to be done to validate the prognostic values of PRDX2 and PRDX4 in ovarian cancer.

## Conclusion

In summary, by using publicly available data from the HPA database, we found that the protein expression of PRDXs family in ovarian cancer tissues was elevated compared with normal ovarian tissues, implying that these proteins may contribute to the progression of ovarian cancer. We also conducted further analysis via KM plotter database and demonstrated that high levels of PRDX3, PRDX5, and PRDX6 predicted an unsatisfactory prognosis in ovarian cancer, and PRDX3 predicted a poor clinical outcome particularly in poor differentiation and late-stage serous ovarian cancer patients. However, the prognostic value of PRDX1, PRDX2, and PRDX4 in ovarian cancer requires further exploration. These results indicate that there are distinct prognostic values of PRDX family members in patients with ovarian cancer, and that the expression of PRDX3, PRDX5, and PRDX6 mRNAs is closely associated with prognostic predictors of the effect of chemotherapy in ovarian cancer patients. Although our results were statistically significant, further studies using larger sample sizes are required to validate these findings and to explore the clinical application of the PRDX family in the treatment of ovarian cancer.

## Abbreviations

CI: confidence interval
HPA: Human Protein Atlas
HR: hazard ratio
IHC: immunohistochemistry
KM plotter: Kaplan–Meier plotter
OS: overall survival
PFS: progression-free survival
PRDXs: peroxiredoxins
ROS: reactive oxygen species

## References

[1] Siegel R.L., Miller K.D. and Jemal A. (2017) Cancer Statistics, 2017. CA Cancer J. Clin. 67, 7–30 10.3322/caac.21387 28055103

[2] Grann A.F. (2011) Survival of patients with ovarian cancer in central and northern Denmark, 1998-2009. Clin. Epidemiol. 3, 59–64 10.2147/CLEP.S20621 21814472 PMC3144780

[3] Klangsin S. (2013) Comparison of the five sonographic morphology scoring systems for the diagnosis of malignant ovarian tumors. Gynecol. Obstet. Invest. 76, 248–253 10.1159/000355563 24192793

[4] Coleman M.P. (2011) Cancer survival in Australia, Canada, Denmark, Norway, Sweden, and the UK, 1995-2007 (the International Cancer Benchmarking Partnership): an analysis of population-based cancer registry data. Lancet 377, 127–138 10.1016/S0140-6736(10)62231-3 21183212 PMC3018568

[5] Jin D.Y. (1997) Regulatory role for a novel human thioredoxin peroxidase in NF-kappaB activation. J. Biol. Chem. 272, 30952–30961 10.1074/jbc.272.49.30952 9388242

[6] Ishii T. (1993) Cloning and characterization of a 23-kDa stress-induced mouse peritoneal macrophage protein. J. Biol. Chem. 268, 18633–18636 8360158

[7] Bae Y.S. (2011) Regulation of reactive oxygen species generation in cell signaling. Mol. Cells 32, 491–509 10.1007/s10059-011-0276-3 22207195 PMC3887685

[8] Ray P.D., Huang B.W. and Tsuji Y. (2012) Reactive oxygen species (ROS) homeostasis and redox regulation in cellular signaling. Cell. Signal. 24, 981–990 10.1016/j.cellsig.2012.01.008 22286106 PMC3454471

[9] Immenschuh S. and Baumgart-Vogt E. (2005) Peroxiredoxins, oxidative stress, and cell proliferation. Antioxid. Redox Signal. 7, 768–777 10.1089/ars.2005.7.768 15890023

[10] Hofmann B., Hecht H.J. and Flohe L. (2002) Peroxiredoxins. Biol. Chem. 383, 347–364 10.1515/BC.2002.040 12033427

[11] Rhee S.G., Chae H.Z. and Kim K. (2005) Peroxiredoxins: a historical overview and speculative preview of novel mechanisms and emerging concepts in cell signaling. Free Radic. Biol. Med. 38, 1543–1552 10.1016/j.freeradbiomed.2005.02.026 15917183

[12] Han S. (2012) Expression and prognostic significance of human peroxiredoxin isoforms in endometrial cancer. Oncol. Lett. 3, 1275–1279 10.3892/ol.2012.648 22783432 PMC3392563

[13] Karihtala P. (2003) Peroxiredoxins in breast carcinoma. Clin. Cancer Res. 9, 3418–3424 12960131

[14] Wu X.Y., Fu Z.X. and Wang X.H. (2010) Peroxiredoxins in colorectal neoplasms. Histol. Histopathol. 25, 1297–1303 20712014 10.14670/HH-25.1297

[15] Basu A. (2011) Differential expression of peroxiredoxins in prostate cancer: consistent upregulation of PRDX3 and PRDX4. Prostate 71, 755–765 10.1002/pros.21292 21031435 PMC3107902

[16] Kim K. (2009) Expression of human peroxiredoxin isoforms in response to cervical carcinogenesis. Oncol. Rep. 21, 1391–1396 19424615

[17] Noh D.Y. (2001) Overexpression of peroxiredoxin in human breast cancer. Anticancer Res. 21, 2085–2090 11497302

[18] Park M.H. (2016) Roles of peroxiredoxins in cancer, neurodegenerative diseases and inflammatory diseases. Pharmacol. Ther. 163, 1–23 10.1016/j.pharmthera.2016.03.018 27130805 PMC7112520

[19] Yanagawa T. (2000) Peroxiredoxin I expression in oral cancer: a potential new tumor marker. Cancer Lett. 156, 27–35 10.1016/S0304-3835(00)00434-1 10840156

[20] Lee E.Y., Kang J.Y. and Kim K.W. (2015) Expression of cyclooxygenase-2, peroxiredoxin I, peroxiredoxin 6 and nuclear factor-kappaB in oral squamous cell carcinoma. Oncol. Lett. 10, 3129–3136 10.3892/ol.2015.3705 26722300 PMC4665682

[21] Yonglitthipagon P. (2012) Prognostic significance of peroxiredoxin 1 and ezrin-radixin-moesin-binding phosphoprotein 50 in cholangiocarcinoma. Hum. Pathol. 43, 1719–1730 10.1016/j.humpath.2011.11.021 22446018 PMC3386378

[22] Zhou J. (2015) Overexpression of Prdx1 in hilar cholangiocarcinoma: a predictor for recurrence and prognosis. Int. J. Clin. Exp. Pathol. 8, 9863–9874 26617696 PMC4637781

[23] Isohookana J. (2016) Loss of peroxiredoxin expression is associated with an aggressive phenotype in pancreatic adenocarcinoma. Anticancer Res. 36, 427–433 26722077

[24] Cai C.Y. (2015) Expression and clinical value of peroxiredoxin-1 in patients with pancreatic cancer. Eur. J. Surg. Oncol. 41, 228–235 10.1016/j.ejso.2014.11.037 25434328

[25] Ji D. (2013) Prognostic role of serum AZGP1, PEDF and PRDX2 in colorectal cancer patients. Carcinogenesis 34, 1265–1272 10.1093/carcin/bgt056 23393224

[26] Peng L. (2017) Peroxiredoxin 2 is associated with colorectal cancer progression and poor survival of patients. Oncotarget 8, 15057–15070 28125800 10.18632/oncotarget.14801 PMC5362467

[27] Woolston C.M. (2011) Expression of thioredoxin system and related peroxiredoxin proteins is associated with clinical outcome in radiotherapy treated early stage breast cancer. Radiother. Oncol. 100, 308–313 10.1016/j.radonc.2011.05.029 21641069

[28] Chung K.H. (2010) Proteomic identification of overexpressed PRDX 1 and its clinical implications in ovarian carcinoma. J. Proteome Res. 9, 451–457 10.1021/pr900811x 19902980

[29] Karihtala P. (2009) DNA adduct 8-hydroxydeoxyguanosine, a novel putative marker of prognostic significance in ovarian carcinoma. Int. J. Gynecol. Cancer 19, 1047–1051 10.1111/IGC.0b013e3181ad0f0d 19820366

[30] Lindskog C. (2016) The Human Protein Atlas - an important resource for basic and clinical research. Expert Rev. Proteomics 13, 627–629 10.1080/14789450.2016.1199280 27276068

[31] Asplund A. (2012) Antibodies for profiling the human proteome-The Human Protein Atlas as a resource for cancer research. Proteomics 12, 2067–2077 10.1002/pmic.201100504 22623277

[32] Thapa S. (2018) Significance of aquaporins’ expression in the prognosis of gastric cancer. Biosci. Rep. 38, BSR20171687, 10.1042/BSR20171687 29678898 PMC5997799

[33] Gyorffy B. (2010) An online survival analysis tool to rapidly assess the effect of 22,277 genes on breast cancer prognosis using microarray data of 1,809 patients. Breast Cancer Res. Treat. 123, 725–731 10.1007/s10549-009-0674-9 20020197

[34] Gyorffy B., Lanczky A. and Szallasi Z. (2012) Implementing an online tool for genome-wide validation of survival-associated biomarkers in ovarian-cancer using microarray data from 1287 patients. Endocr. Relat. Cancer 19, 197–208 10.1530/ERC-11-0329 22277193

[35] Gyorffy B. (2013) Online survival analysis software to assess the prognostic value of biomarkers using transcriptomic data in non-small-cell lung cancer. PLoS One 8, e82241 10.1371/journal.pone.0082241 24367507 PMC3867325

[36] Perkins A. (2015) Peroxiredoxins: guardians against oxidative stress and modulators of peroxide signaling. Trends Biochem. Sci. 40, 435–445 10.1016/j.tibs.2015.05.001 26067716 PMC4509974

[37] Song I.S. (2011) Mitochondrial peroxiredoxin III is a potential target for cancer therapy. Int. J. Mol. Sci. 12, 7163–7185 10.3390/ijms12107163 22072940 PMC3211031

[38] Egler R.A. (2005) Regulation of reactive oxygen species, DNA damage, and c-Myc function by peroxiredoxin 1. Oncogene 24, 8038–8050 10.1038/sj.onc.1208821 16170382

[39] Cao J. (2009) Prdx1 inhibits tumorigenesis via regulating PTEN/AKT activity. EMBO J. 28, 1505–1517 10.1038/emboj.2009.101 19369943 PMC2688529

[40] Wang X. (2010) Selective association of peroxiredoxin 1 with genomic DNA and COX-2 upstream promoter elements in estrogen receptor negative breast cancer cells. Mol. Biol. Cell 21, 2987–2995 10.1091/mbc.e10-02-0160 20631257 PMC2929992

[41] Jiang L. (2014) Proteomic analysis of bladder cancer indicates Prx-I as a key molecule in BI-TK/GCV treatment system. PLoS One 9, e98764 10.1371/journal.pone.0098764 24904997 PMC4048271

[42] Zhang M. (2014) Induction of peroxiredoxin 1 by hypoxia regulates heme oxygenase-1 via NF-kappaB in oral cancer. PLoS One 9, e105994 10.1371/journal.pone.0105994 25162226 PMC4146557

[43] Hwang K.E. (2013) Elevated prx1 provides resistance to docetaxel, but is not associated with predictive significance in lung cancer. Tuberc. Respir. Dis. 75, 59–66 10.4046/trd.2013.75.2.59 24023558 PMC3766810

[44] Gong F. (2015) Peroxiredoxin 1 promotes tumorigenesis through regulating the activity of mTOR/p70S6K pathway in esophageal squamous cell carcinoma. Med. Oncol. 32, 455 10.1007/s12032-014-0455-0 25579166

[45] O’Leary P.C. (2014) Peroxiredoxin-1 protects estrogen receptor alpha from oxidative stress-induced suppression and is a protein biomarker of favorable prognosis in breast cancer. Breast Cancer Res. 16, R79 10.1186/bcr3691 25011585 PMC4226972

[46] Hoshino I. (2007) Tumor suppressor Prdx1 is a prognostic factor in esophageal squamous cell carcinoma patients. Oncol. Rep. 18, 867–871 17786348

[47] Kim J.H. (2008) Up-regulation of peroxiredoxin 1 in lung cancer and its implication as a prognostic and therapeutic target. Clin. Cancer Res. 14, 2326–2333 10.1158/1078-0432.CCR-07-4457 18413821

[48] Sun Q.K. (2014) Diagnostic and prognostic significance of peroxiredoxin 1 expression in human hepatocellular carcinoma. Med. Oncol. 31, 786 10.1007/s12032-013-0786-2 24297309

[49] Chen M.F. (2010) Role of peroxiredoxin I in rectal cancer and related to p53 status. Int. J. Radiat. Oncol. Biol. Phys. 78, 868–878 10.1016/j.ijrobp.2010.05.025 20732753

[50] Lu W. (2014) Peroxiredoxin 2 knockdown by RNA interference inhibits the growth of colorectal cancer cells by downregulating Wnt/beta-catenin signaling. Cancer Lett. 343, 190–199 10.1016/j.canlet.2013.10.002 24125860

[51] Shiota M. (2011) Peroxiredoxin 2 in the nucleus and cytoplasm distinctly regulates androgen receptor activity in prostate cancer cells. Free Radic. Biol. Med. 51, 78–87 10.1016/j.freeradbiomed.2011.04.001 21539911

[52] Raatikainen S. (2015) Increased Peroxiredoxin 6 Expression Predicts Biochemical Recurrence in Prostate Cancer Patients After Radical Prostatectomy. Anticancer Res. 35, 6465–6470 26637857

[53] Soini Y. (2006) Oxidative/nitrosative stress and peroxiredoxin 2 are associated with grade and prognosis of human renal carcinoma. APMIS 114, 329–337 10.1111/j.1600-0463.2006.apm_315.x 16725008

[54] Jarvela S. (2010) Specific expression profile and prognostic significance of peroxiredoxins in grade II-IV astrocytic brain tumors. BMC Cancer 10, 104 10.1186/1471-2407-10-104 20307276 PMC2858108

[55] Sova H. (2012) Down-regulation of 8-hydroxydeoxyguanosine and peroxiredoxin II in the pathogenesis of endometriosis-associated ovarian cancer. Anticancer Res. 32, 3037–3044 22843871

[56] Li X.Q. (2009) Proteomic identification of tumor-associated protein in ovarian serous cystadenocarinoma. Cancer Lett. 275, 109–116 10.1016/j.canlet.2008.10.019 19056166

[57] Chua P.J. (2010) Silencing the peroxiredoxin III gene inhibits cell proliferation in breast cancer. Int. J. Oncol. 36, 359–364 20043069

[58] Hintsala H.R. (2015) Dysregulation of redox-state-regulating enzymes in melanocytic skin tumours and the surrounding microenvironment. Histopathology 67, 348–357 10.1111/his.12659 25627040

[59] Shi L. (2014) Serum peroxiredoxin3 is a useful biomarker for early diagnosis and assessemnt of prognosis of hepatocellular carcinoma in Chinese patients. Asian Pac. J. Cancer Prev. 15, 2979–2986 10.7314/APJCP.2014.15.7.2979 24815434

[60] Duan J. (2013) siRNA targeting of PRDX3 enhances cisplatininduced apoptosis in ovarian cancer cells through the suppression of the NFkappaB signaling pathway. Mol. Med. Rep. 7, 1688–1694 10.3892/mmr.2013.1370 23503975

[61] Wang X.Y., Wang H.J. and Li X.Q. (2013) Peroxiredoxin III protein expression is associated with platinum resistance in epithelial ovarian cancer. Tumour Biol. 34, 2275–2281 10.1007/s13277-013-0769-0 23564483

[62] Wei Q. (2011) Sulfiredoxin-Peroxiredoxin IV axis promotes human lung cancer progression through modulation of specific phosphokinase signaling. Proc. Natl. Acad. Sci. U.S.A. 108, 7004–7009 10.1073/pnas.1013012108 21487000 PMC3084097

[63] Yi N. (2014) High expression of peroxiredoxin 4 affects the survival time of colorectal cancer patients, but is not an independent unfavorable prognostic factor. Mol. Clin. Oncol. 2, 767–772 10.3892/mco.2014.317 25054044 PMC4106752

[64] Hwang J.A. (2015) Peroxiredoxin 4 as an independent prognostic marker for survival in patients with early-stage lung squamous cell carcinoma. Int. J. Clin. Exp. Pathol. 8, 6627–6635 26261544 PMC4525878

[65] Chang K.P. (2011) Identification of PRDX4 and P4HA2 as metastasis-associated proteins in oral cavity squamous cell carcinoma by comparative tissue proteomics of microdissected specimens using iTRAQ technology. J. Proteome Res. 10, 4935–4947 10.1021/pr200311p 21859152

[66] Soini Y. (2011) 8-hydroxydeguanosine and nitrotyrosine are prognostic factors in urinary bladder carcinoma. Int. J. Clin. Exp. Pathol. 4, 267–275 21487522 PMC3071659

[67] Gerard A.C. (2005) Peroxiredoxin 5 expression in the human thyroid gland. Thyroid 15, 205–209 10.1089/thy.2005.15.205 15785239

[68] Kim B. (2017) Peroxiredoxin 5 overexpression enhances tumorigenicity and correlates with poor prognosis in gastric cancer. Int. J. Oncol. 51, 298–306 10.3892/ijo.2017.4013 28535004

[69] Xu X. (2016) The phospholipase A2 activity of peroxiredoxin 6 promotes cancer cell death induced by tumor necrosis factor alpha in hepatocellular carcinoma. Mol. Carcinog. 55, 1299–1308 10.1002/mc.22371 26293541

[70] Yun H.M. (2015) PRDX6 promotes tumor development via the JAK2/STAT3 pathway in a urethane-induced lung tumor model. Free Radic. Biol. Med. 80, 136–144 10.1016/j.freeradbiomed.2014.12.022 25582888

[71] Yun H.M. (2014) PRDX6 promotes lung tumor progression via its GPx and iPLA2 activities. Free Radic. Biol. Med. 69, 367–376 10.1016/j.freeradbiomed.2014.02.001 24512906

[72] Kuusisto M.E. (2015) High intensity of cytoplasmic peroxiredoxin VI expression is associated with adverse outcome in diffuse large B-cell lymphoma independently of International Prognostic Index. J. Clin. Pathol. 68, 552–556 10.1136/jclinpath-2014-202771 25935550

[73] Klaunig J.E. (1998) The role of oxidative stress in chemical carcinogenesis. Environ. Health Perspect. 106, 289–295 10.1289/ehp.98106s1289 9539021 PMC1533298

[74] Pak J.H. (2011) Peroxiredoxin 6 overexpression attenuates cisplatin-induced apoptosis in human ovarian cancer cells. Cancer Invest. 29, 21–28 10.3109/07357907.2010.535056 21166495

[75] Kalinina E.V. (2012) Expression of peroxiredoxin 1, 2, 3, and 6 genes in cancer cells during drug resistance formation. Bull. Exp. Biol. Med. 153, 878–881 10.1007/s10517-012-1849-7 23113308

[76] Cruz I.N. (2017) Proteomics analysis of ovarian cancer cell lines and tissues reveals drug resistance-associated proteins. Cancer Genomics Proteomics 14, 35–51 10.21873/cgp.20017 28031236 PMC5267499

[77] Sehrawat U. (2016) Comparative proteomic analysis of advanced ovarian cancer tissue to identify potential biomarkers of responders and nonresponders to first-line chemotherapy of carboplatin and paclitaxel. Biomark Cancer 8, 43–56 10.4137/BIC.S35775 26997873 PMC4795487

